# Microglial pyroptosis: Therapeutic target in secondary brain injury following intracerebral hemorrhage

**DOI:** 10.3389/fncel.2022.971469

**Published:** 2022-09-09

**Authors:** Lingui Gu, Mingjiang Sun, Ruihao Li, Yihao Tao, Xu Luo, Xingyu Zhang, Ye Yuan, Zongyi Xie

**Affiliations:** Department of Neurosurgery, The Second Affiliated Hospital of Chongqing Medical University, Chongqing, China

**Keywords:** intracerebral hemorrhage, secondary brain injury, microglia, pyroptosis, neuroinflammation

## Abstract

Intracerebral hemorrhage (ICH) is a major cerebrovascular illness that causes substantial neurological sequelae and dysfunction caused by secondary brain injury (SBI), and there are no effective therapies to mitigate the disability. Microglia, the brain-resident macrophage, participates in the primary inflammatory response, and activation of microglia to an M1-like phenotype largely takes place in the acute phase following ICH. A growing body of research suggests that the pathophysiology of SBI after ICH is mediated by an inflammatory response mediated by microglial-pyroptotic inflammasomes, while inhibiting the activation of microglial pyroptosis could suppress the inflammatory cascade reaction, thus attenuating the brain injury after ICH. Pyroptosis is characterized by rapid plasma membrane disruption, followed by the release of cellular contents and pro-inflammatory mediators. In this review, we outline the molecular mechanism of microglial pyroptosis and summarize the up-to-date evidence of its involvement in the pathological process of ICH, and highlight microglial pyroptosis-targeted strategies that have the potential to cure intracerebral hemorrhage. This review contributes to a better understanding of the function of microglial pyroptosis in ICH and assesses it as a possible therapeutic target.

## Introduction

Intracerebral hemorrhage (ICH) is an atraumatic-spontaneous hemorrhage of the brain parenchyma, which has the highest mortality rate among cerebrovascular diseases (CVD) (An et al., 2017). The main factor contributing to the poor prognosis of ICH is secondary brain injury (SBI), which is characterized by a complex array of pathologies, such as extensive neuroinflammation caused by enlargement of hematoma and changes of metabolite, oxidative stress, and neurotoxicity induced by the release of blood components, programmed cell death, rupture of blood-brain-barrier (BBB), increase of brain edema around hematoma (Weimar and Kleine-Borgmann, 2017; Garg and Biller, 2019; Nobleza, 2021). Microglia are essential innate immune cells that serve as the brain’s guardians and are considered as the first non-neuronal cells to react to several types of acute brain injuries, including ICH (Wang, 2010; Lan et al., 2011). As the primary phagocytes of the brain, microglia generate either classically activated (M1, proinflammatory) or activated (M2, anti-inflammatory) phenotypes, a process known as polarization (Taylor and Sansing, 2013; Xiong et al., 2016). Despite an increase in global M1 and M2 marker levels during the acute phase, flow cytometry revealed that macrophages and microglia mostly formed an M1 phenotype (Lan et al., 2017). The applicability of these putative activation states to macrophage activity *in vivo* is debatable, despite markers of M1 and M2 microglia and macrophage polarization may be easily identified. The signaling mechanisms that result in subsequent damage following ICH are very intricate. Investigating transcription factor activity in response to brain damage is thus critical to our knowledge of the microglial M1 to M2 transition following ICH. However, relatively few researchers have looked into transcription factors other than NF-kB following ICH (Hume, 2015). Proinflammatory cytokines are often produced when microglia are activated. Currently, it is believed that only classically active M1 microglia generate proinflammatory cytokines and that changes in proinflammatory cytokine levels and profiles account for the changes in microglial function following ICH (Zhang et al., 2014; Greter et al., 2015). In the meantime, inflammasome activation-induced pyroptosis occurred in microglia.

Pyroptosis is a novel type of programmed cell death that relies on the cysteine-dependent aspartate-specific protease (caspase) family. The assembly and activation of the inflammasome resulted in the activation of numerous caspases, which then encouraged the cleavage of Gasdermin family members (GSDMs), generated active amino-terminal, migrated to the cell membrane to perforate, causing cellular infiltration and swelling, and ruptured the cell membrane, releasing a significant amount of inflammatory factors and other cytoplasmic contents (Cookson and Brennan, 2001; Chen et al., 2018). Pyroptosis is thought to be a significant inflammatory-effector mechanism induced by aseptic inflammation after ICH, promoting the synthesis and release of interleukins and pro-inflammatory factors, which in turn triggers and amplifies inflammatory responses, and results in additional harm to brain tissues and cells (Zhaolin et al., 2019; Fang et al., 2020). Numerous studies in recent years have shown microglial caspase-mediated pyroptosis could wreak havoc on neurological performance and neurology function after ICH, and inhibition of microglial pyroptosis could alleviate brain injury and neurological function, which indicated that regulation of microglial pyroptosis might be a vital therapeutic target after ICH (Chen et al., 2019c; Xu et al., 2021a; Gu et al., 2022).

## Pyroptosis and pyroptotic-associated protein

### Inflammasome

The inflammasome is an intracellular multiprotein complex, assembled by specific germline coding Pattern Recognition Receptors (PRRs). These receptors could detect abnormal cellular environments *via* recognizing Danger Associated Molecular Patterns (DAMPs) and Pathogen-Associated Molecular Patterns (PAMPs). PRRs are divided into two categories based on subcellular localization: Transmembrane Toll-like receptors (TLR) and C-type Lectin receptors (CLRs) are located in the plasma membrane and endosomes, and cytoplasmic proteins are located in the intracellular compartments, such as Retinoic acid-inducible gene (RIG) -I like receptors (RLRs), Absent in Melanoma-like receptors (AIM2) and Nod-like receptor Pyrin domain-containing (NLR) (Vande Walle and Lamkanfi, 2016; Barrington et al., 2017; Liang et al., 2020).

Generally, PRRs (NLRs or ALRs), which act as sensors, interact with pyrin domains after DAMP or PAMP is recognized, and recruit apoptosis-associated speck-like protein containing (ASC) which included caspase recruitment domains, CARD, and then ASC oligomerized to form large spots polymer, which recruits effector molecules – caspase precursors through C-terminal CARD, and then assemble into inflammasome complexes. The interaction of CARD-CARD activates the caspase precursor, which then cleaves GSDMs and the pro-interleukin-1β (pro-IL-1β) and pro-IL-18, triggering pyroptosis (Bergsbaken et al., 2009; Schroder and Tschopp, 2010). In general, NLRs and ALRs are common sensor molecules in inflammatory activation, especially NLRs (Martinon et al., 2002; Lamkanfi and Dixit, 2014). NLR belongs to PRR that can be activated by not only exogenous but endogenous activators (Howrylak and Nakahira, 2017). Currently, four NLRs (NLRP1, NLRP3, NLRP6, and NLRC4) have been reported to involve in the assembly and formation of inflammasomes (Man and Kanneganti, 2015; Howrylak and Nakahira, 2017). Except for NLRP1, NLR typically contains three domains: the N-terminal junction domain [e.g., CARD or Pyrin domain (PYD)], the central nucleotide domain (NBD), and the C-terminal leucine-rich repeat sequence (LRR) domain. N-terminal domain is responsible for the recruitment of CARD and interaction of CARD-CARD, which activate caspase precursors. NBD is critical for ASC oligomerization, and LRR is mainly used for sensing bacterial components (Shaw et al., 2008; Franchi et al., 2009).

**FIGURE 1:**
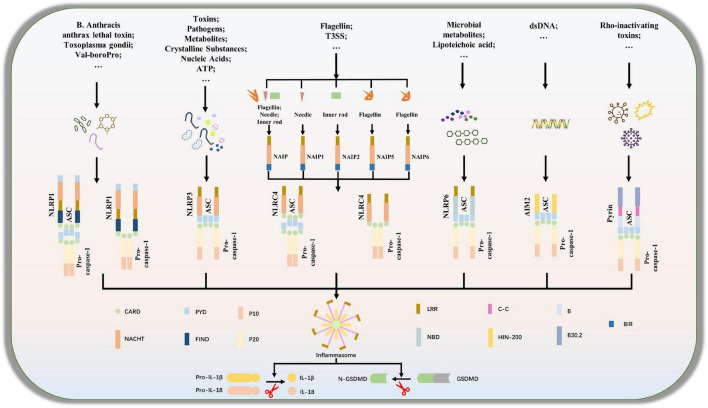
Composition of inflammasome complexes. A subset of NOD-like receptors (NLRs), Nlrp1,Nlrp3, Nlrp6 and Nlrc4, as well as the cytosolic DNA sensor absent in melanoma 2, AIM2 and the protein product of Mediterranean fever (MEFV), Pyrin is known to engage well-defined inflammasomes to which caspase-1 is recruited directly or through the adaptor protein apoptosis-associated speck-like protein containing a CARD (ASC).

Although NLRP3 in the NLRs family is the most characteristic inflammatory body sensor, numerous additional sensors, shown in Figure 1, including NLRP1, NLRC4, NLRP6, and AIM2 in ALRs, can also generate inflammasomes and engage in the control of host immunological and inflammatory responses (Broz and Dixit, 2016; Kay et al., 2020). In ICH, NLRP1, NLRP3, NLRP6, NLRC4, etc., are mostly studied, as shown in Supplementary Table 1 (Yao et al., 2017; Chen et al., 2019c; Xiao et al., 2020; Gan et al., 2021; Yan et al., 2021). Then, we’ll go through various inflammasome subtypes that were present in ICH.

### Caspase family

The highly conserved cysteine protease family, which includes caspase, is important for both inflammation and programmed cell death (Galluzzi et al., 2016; Man and Kanneganti, 2016). The caspase family has similar protein structures: an amino end that connects CARD and a carboxyl end. According to the structure and function of the caspase domain, mammalian caspases can be divided into three subclasses: inflammatory caspase-1, 4, 5, 12, 13, 14, initiator caspase-2, 8, 9, 10, and executioner caspase-3, 6, 7 (Chowdhury et al., 2008; Man and Kanneganti, 2016; Kesavardhana and Kanneganti, 2017). In multicellular organisms, caspases are activated by macromolecular complexes that bring inactive pro-caspases together and promote their adjacent-induced self-activation and proteolytic processing. Specific caspase activation ultimately leads to particular types of programmed cell death (Alnemri et al., 1996). In previous studies, Nagata’s team has found that apoptosis-related caspase resulting in apoptosis is a non-inflammatory process (Nagata, 2018), but recent studies have found that apoptotic caspases and inflammatory caspases can crosstalk and regulate each other. Inflammatory caspase-1 initiates apoptosis in GSDMD deficient cells *via* the Bid-caspase-9-caspase-3 axis, and caspase-8 cleaves GSDMD during Yersinia infection to induce pyroptosis. Chemotherapy drugs or apoptotic inducers can activate caspase-3 to cleave GSDME (DFNA5) and induce pyroptosis (Rogers et al., 2017; Wang et al., 2017; Sarhan et al., 2018; Tsuchiya et al., 2019).

#### Inflammatory-related caspases

The control of pro-inflammatory variables depends heavily on caspase-1, the most extensively researched and representative inflammatory caspase (Martinon and Tschopp, 2007). Its main function is to activate pro-IL-1β and pro-IL-18 to increase the inflammatory. Additionally, It also cleaves GSDMD to cause pyroptosis through the classical-pyroptotic pathway (Shi et al., 2017). Caspase-1 expression increased following ICH (D’Osualdo et al., 2011; Liang et al., 2019). Caspase-1 inhibition reduced the protein level of GSDMD-NT, relieved brain damage, and improved neurological function (Liang et al., 2019; Gu et al., 2022). These findings indicate that caspase-1 plays a role in the causes of brain damage caused by ICH-mediated pyroptosis.

Caspase-4,5,11, which mediates non-classical pyroptosis, is mainly expressed in human macrophages, monocytes, epithelial cells, and keratinocytes, while caspase-11 is mainly expressed in mice. Unlike caspase-1, which requires the assembly of inflammasomes, caspase-11 could directly detect cytoplasmic lipopolysaccharide (LPS) and mediate inflammatory cell death after intracellular bacterial infection (Kayagaki et al., 2013; Shi et al., 2014; Matikainen et al., 2020). LPS is a key factor in the activation of caspase-4/5/11 and activated caspase-4/5/11 can form non-selective channels, leading to cell pyrosis through cleavage of GSDMD to perforate cell membranes (Kayagaki et al., 2015). In recent studies, Seo et al’s team and Lei et al’s team have found that caspase-4/5/11 also activates the channel of Pannexin-1 to regulate the secretion of inflammatory mediators into the extracellular domain (Lei et al., 2021; Seo et al., 2021). In the I/R model of rats, caspase-11 was mainly expressed in microglia and astrocytes, and pro-cleaved caspase-11, GSDMD-FL/NT, NLRP3, IL-6, and IL-1β were significantly increased (Lyu et al., 2021). Caspase-11 can directly induce pyroptosis by detecting LPS and indirectly activate non-canonical NLRP3 inflammasome, IL-18, and IL-1β, whereas Il-1 β reversely stimulates the caspase-11 expression during aseptic inflammation (Shi et al., 2014; Caution et al., 2019).

#### Apoptosis-related caspase

Caspase-2, 3, 6, 7, 8, 9, and 10 are apoptosis-related caspases, which were further divided into initiator caspase (caspase-8, -9, and -10) and executioner caspase (caspase-3, -6, and -7) (Man and Kanneganti, 2016). Caspase-3 has long been considered a key marker of cell apoptosis, but recent studies have shown that caspase-3 can cleave GSDME to form N-terminal domains that recognize and punch holes in the cell membrane, causing cell swelling and rupture, releasing inflammatory factors and damage-associated molecular patterns (Wang et al., 2017). Caspase-8 is an apoptotic caspase, yet it can promote the maturation of IL-1β and participate in inflammatory cell death (Maelfait et al., 2008; Rickard et al., 2014). Pathogenic Yersinia can inhibit TAK1 function by effector YopJ, inactivating TAKI, and activating RIPK1 and dependent cleavage of caspase-8 to cleave GSDMD (GSDME in mouse macrophages) (Orning et al., 2018; Sarhan et al., 2018). In addition to Caspase-1 or Caspase-4/-5/-11, Caspase-8 also directly regulates the cleavage and activation of gasdermin protein to induce pyroptosis (Sarhan et al., 2018), but the efficiency of caspase-8 in cleaving GSDMD is much lower than that of caspase-1 (Chen et al., 2019a). The effect and mechanism of apoptosis-related caspase on ICH-induced pyroptosis remains unclear.

#### Others

Both caspase-2-dependent apoptosis and autophagy are considered to be alternative cell death pathways in casp1^–/–^ macrophages, but the relationship with ICH-induced pyroptosis has not been explored (Jesenberger et al., 2000; Hernandez et al., 2003). Caspase-6 has been shown to cleave Lamin A to promote chromosomal cohesion during the execution of apoptosis, and its dysregulation has been observed in inflammatory neurodegenerative diseases such as Huntington’s disease and Alzheimer’s disease. However, its role in infection-induced cell death and the specific upstream signaling pathways remain unclear (Guo et al., 2004; Wang et al., 2015). Although caspase-12 is also an inflammatory caspase, which seems to mediate endoplasmic reticulum (ER) stress-induced apoptosis, and negatively regulates inflammation by promoting degradation of NF-κB-induced kinase (NIK) (Kalai et al., 2003; Di Sano et al., 2006). Caspase-12 is highly expressed in intestinal tissues and plays an important role in maintaining intestinal inflammation and tumorigenesis, but there is still no research on ICH-induced pyroptosis. Caspase-14 is known to be involved in epidermal differentiation, but its role in pyroptosis is still unclear (Eckhart et al., 2000; Lippens et al., 2000).

### Gasdermin family

The gasdermin protein family consists of GSDMA, GSDMB, GSDMC, GSDMD, GSDME (DFNA5), and autosomal-recessive non-syndromic hearing impairment type 59 (DFNB59). These proteins have similar N-terminal and C-terminal domain structures, with the N-terminal containing an effector domain punching membrane holes and performing pyroptosis through its pore-forming activity, while the C-terminal domain acts as an inhibitory domain to balance N-terminal function (Van Laer et al., 1998; Delmaghani et al., 2006; Saeki et al., 2009; Miguchi et al., 2016; Chen et al., 2019b). However, the residues of caspase cleavage sites in the junction region are different among members of the gasdermin family, and not all gasdermin proteins can be cleaved by caspase, only the GSDMD junction region is cleaved by inflammatory caspase. Further study showed that caspase-3 cleaved GSDME in the junction region to release the N-terminal fragment, which activated pyroptosis by a similar mechanism as GSDMD-N. Caspase-3 also cleaves GSDMD, however, this cleavage occurs in the N-terminal domain rather than in the junction region, which inhibits its function (Wang et al., 2017). In addition to DFNB59, the gasdermin protein family plays different roles in cell pyroptosis (Feng et al., 2018).

Gasdermin D is the primary executor of pyroptosis and acts as a downstream effector in both canonical and non-canonical pyroptotic pathways in a variety of neurological disease models, including ICH. In recent studies, Fann and his team reported that GSDME-NT can also punch holes in the membrane to mediate pyroptosis. In the I/R model of the CNS, caspase-3 cleaved GSDME into GSDME-NT, and the GSDME-NT oligomerized to form holes in the plasma membrane, releasing IL-1β and IL-18 to induce secondary cell pyroptosis and brain injury (Fann et al., 2020). GSDME is mainly expressed in the brain, female genital tract (especially placenta), kidney, and muscle, but its specific mechanism in the brain remains unclear and needs further exploration (Van Laer et al., 1998). GSDMA is mainly associated with autoimmune diseases and cancer (Saeki et al., 2009), and GSDMB is lysed by lymphocyte-derived Granzymes A (GZMA), activating its pore-forming activity and inducing pyroptosis (Zhou et al., 2020). GSDMB is associated with autoimmunities such as asthma and inflammatory bowel disease (Chao et al., 2017). Meanwhile, it has been reported that GSDMB overexpression is associated with breast cancer (Hergueta-Redondo et al., 2016), gastric cancer (Komiyama et al., 2010), and cervical cancer (Sun et al., 2008). GSDMC is highly expressed in the gastrointestinal tract (Miguchi et al., 2016). DFNB59 is associated with non-comprehensive deafness (Delmaghani et al., 2006).

## Pyroptosis-related mechanism pathways

**FIGURE 2:**
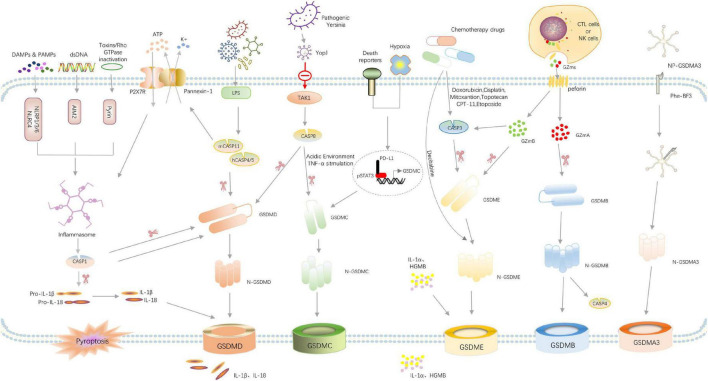
Overview of signaling pathways that mediate pyroptosis. Recognition of pathogen-associated molecular patterns (PAMPs) or danger-associated molecular patterns (DAMPs) or double-stranded DNA (dsDNA) by their respective inflammasome-sensors NLRP1/3/6, NLRC4, AIM2, and Pyrin leads to the assembly of a multi-protein complex termed the inflammasome. Consequently, caspase-1 undergoes autoproteolytic processing to lock the protease in its active form. Caspase-1 directly cleaves its substrates gasdermin D and the pro-inflammatory cytokines pro-IL-1β and pro-IL-18. The N-terminal cleavage fragment of Gsdmd forms pores in the host cell membrane thereby mediating the release of cytoplasmic content, the mature IL-1β and IL-18 and the other DAMPs IL-1α, HMGB1 and ATP. Cytosolic LPS activates caspase-4/5 and caspase-11, triggering pyroptosis by cleaving GSDMD. The activated caspase-11 also cleaves Pannexin-1, inducing ATP release and P2 × 7R-related pyroptotic cell death. Pathogenic Yersinia can inhibit TAK1 function by effector YopJ, and then induces the activation of caspase-8, which cleaves GSDMD, resulting in pyroptosis. In addition, under hypoxia conditions, PD-L1 is transferred to the nucleus and regulates the transcription of GSDMC together with p-Stat3, resulting in the conversion of apoptosis to pyroptosis after TNFα-activated caspase-8. Chemotherapy drugs can activate caspase-3 to cleave GSDME and induce pyroptosis. In the granzyme-mediated pathway, CAR T cells rapidly activate caspase-3 in target cells by releasing GzmB, and then GSDME was activated, causing extensive pyroptosis. In addition, GzmA and GzmB in cytotoxic lymphocytes enter target cells through perforin and induce pyroptosis. GzmA hydrolyzes GSDMB, and GzmB directly activates GSDME. The administration of NP-GSDMA3 and Phe-BF3 induces GSMDA3-mediated pyroptosis. GSDMA acts as both a sensor and substrate of GAS SpeB and as an effector to trigger pyroptosis.

The pyroptosis-related pathways were shown in Figure 2.

### Canonical pyroptotic pathway dependent on caspase-1

The canonical pyroptotic pathway is typically due to PAMP and DAMP and cytoplasmic disturbances (recently called dynamic balance altering molecular properties, HAMP) were recognized by PRR, which interacts with each other and recruits ASC, and then ASC oligomerized to recruit pro-caspase-1 to assemble into inflammasomes. activation of pro-caspase-1 triggers to cleave GSDMD, pro-IL-1β and IL-18 into their active form, and GSDMD-NT terminal domain acts on phospholipid molecules of the cellular membrane to form non-selective pores, resulting in cell infiltration, swelling and rupture, causing the release of inflammatory content and cell pyroptosis. In models of ICH, activation of inflammatory bodies (NLRP1, NLRP3, NLRC4) and activation of the pathway of caspase-1/GSDMD-mediated pyroptosis is considered the most common cause of neurological deficits. (Yao et al., 2017; Gan et al., 2021; Yan et al., 2021).

### Non-canonical pyroptotic pathways dependent on caspase-4,5,11

The non-canonical pyroptotic pathway does not require PRR of inflammasome to recognize DAMP or PAMP, but caspase is directly activated by sensing LPS to cut GSDMD and induce pyroptosis. Human caspase-4/5 and mouse caspase-11 can detect LPS directly and act as both the sensor and effector molecules of LPS. It is unknown whether the mechanism of brain injury caused by ICH is through the non-canonical pyroptotic pathway, but studies have shown that caspase-4/5/11 can regulate the secretion of inflammatory mediators into the extracellular by activating the Pannexin-1 pathway (Seo et al., 2021). Pannexin-1 could bind with P2 × 7R and contribute to cell membrane integrity, and this interaction has been determined by co-immunoprecipitation and adjacent junctions (Pelegrin and Surprenant, 2006; Poornima et al., 2012; Boyce and Swayne, 2017). P2 × 7R, acting as an ATP-gated transmembrane cation channel, could activate the NLRP3 inflammasome, and then induce caspase-1/GSDMD-mediated pyroptosis (Feng et al., 2015; Boyce and Swayne, 2017). Therefore, in ICH, brain injury may be caused by crosstalk and combined action between canonical pyroptotic pathway and non-canonical pyroptotic pathway, which is worthy of further study to find more targets for the treatment of ICH.

### Pyroptotic pathways dependent on apoptosis-related caspase

Apoptosis-related caspases, such as caspase-3, 8, can produce GSDME-NT by cutting GSDME, thus punching holes in the cell membrane and causing cell pyroptosis (Wang et al., 2017; Sarhan et al., 2018). Shao’s team found that when treated with chemotherapy drugs, cells with high GSDME expression could transform TNF-α-induced apoptosis into caspase-3 cleaved-pyroptosis (Wang et al., 2017). In the environment of acidic or TNF-α stimulation *in vivo*, caspase-8 cleaved GSDMC specifically to generate N-terminal domain forming holes in the cell membrane and inducing cell pyroptosis (Hou et al., 2020; Zhang et al., 2021).

### Granzymes mediated pathway

Granzymes are a serine protease secreted by natural killer cells (NK) and cytotoxic T lymphocytes (CTL). It enters targeted cells through perforin (PFN) in toxic particles, activates specific intracellular signals, and induces cell death. When granzymes were delivered directly to tumor cells during a cytotoxic T cell attack, GSDMB and GSDME were cleaved into N- and C- terminal fragments by GZmA and GZmB, respectively. Once cleaved, GSDMB and GSDME induce pyroptosis in tumor cells (Zhang et al., 2020b; Zhou et al., 2020). Granzymes (GZms) mediated pyroptosis has been seen as a way to tackle tumors. However, it is still necessary to further study whether and how granzymes-mediated pyroptosis leads to ICH-induced brain injury.

### Others

Wang and colleagues established a biorthogonal system using NP-GSDMA3, which results from the conjugation of gasdermin A3 protein to golden nanoparticles, and the cancer-imaging probe phenylalanine trifluoroborate (Phe-BF3) to investigate the correlation between pyroptosis and immunity. The published evidence indicates that the murine mammary carcinoma cell line 4T1 undergoes pyroptosis after treatment with NP-GSDMA3 and Phe-BF3 (inducing the release of GSDMA3 after NP-GSDMA3 enters cancer cells), and this effect is accompanied by elevated levels of gasdermin N-termini (Pulaski and Ostrand-Rosenberg, 2001; Wang Q. et al., 2020; Hsu et al., 2021). Besides, streptococcus pyogenes is a kind of skin pathogen, known as group A Streptococcus (GAS). Deng et al. found that the GAS cysteine protease streptococcal pyrogenic exotoxin B (SpeB) virulence factor induces pyroptosis in keratinocytes by cleaving GSDMA after Gln246, unleashing an active N-terminal fragment that triggers pyroptosis. GSDMA acts as both a sensor and substrate of GAS SpeB and as an effector to trigger pyroptosis, adding a simple one-molecule mechanism for host recognition and control of virulence of a dangerous microbial pathogen (Deng et al., 2022).

## Microglial pyroptosis inhibitor targets for treating secondary brain injury following intracerebral hemorrhage

**FIGURE 3:**
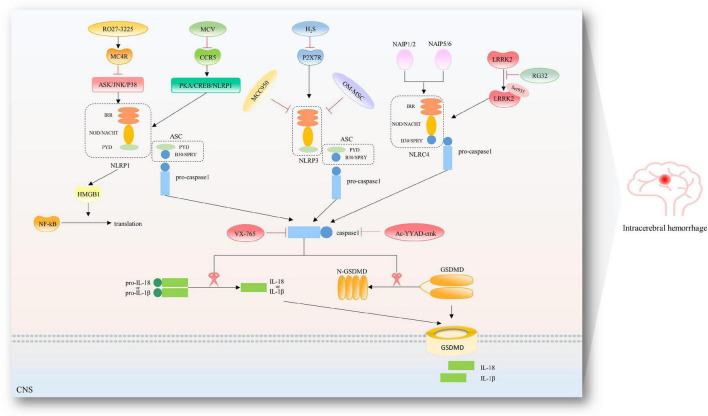
Therapeutic targets in the pyroptotic pathways after ICH. To date, several inhibitors have been described for the NLRP1/NLRP3/NLRC4 inflammasome and caspase-1 after ICH. RO27-3225 suppressed NLRP1-dependent pyroptosis *via* inhibiting MC4R-mediated ASK/JNK/P38 MAPK signaling pathway. Maraviroc (MVC) suppressed PKA/CREB/NLRP1 signaling pathway *via* inhibiting CCR5. Hydrogen sulfide (H_2_S) attenuated NLRP3 inflammasome by inhibiting P2 × 7 receptor. Hypoxia-pretreated olfactory mucosal mesenchymal stem cells (OM-MSCs) are known to decrease the expression of NLRP3 inflammasome. Additionally, specific inhibition of NLRP3 has been reported by studies using its derivative MCC950. RGS2 suppressed the interaction of LRRK2 and NLRC4 and NLRC4 inflammasome activation by regulating pLRRK2. Belnacasan (VX-765) and Ac-YYAD-cmk are bioavailable prodrugs of a potent inhibitor for caspase-1.

Excessive activation of inflammasome and subsequent release of proinflammatory cytokines play a crucial role in microglial pyroptosis-mediated brain injury in ICH, and there are many attempts to inhibit the microglial pyroptosis pathway to alleviate neurological deficits after ICH, shown as in Figure 3.

### Inhibition of NLRP3/caspase-1/gasdermin D

In recent research of ICH model of rats, Liang and his team adopted Ac-Tyr-Val-Ala-Asp-chloromethyl ketone (Ac-YVAD-cmk), an inhibitor of Caspase-1, to inhibit activation of caspase-1 and production and maturation of IL-1β, reduce brain injury and improve neurological deficits, but does not affect the expression of upstream inflammatory complex NLRP3 (Liang et al., 2019). Zhao et al’s team and Zhang et al’s team used endogenous hydrogen sulfide to attenuate NLRP3 inflammasome by inhibiting P2 × 7 receptor after ICH in rats, thereby reducing caspase-1/IL-1β-mediated pyroptosis (Zhao et al., 2017; Zhang et al., 2020a). Li et al’s team found that androgynolide reduces the level of IL-1β and LDH by inhibiting the assembly of the NLRP3 inflammasome, alleviating ICH-induced microglial pyroptosis, thus alleviating neurobehavioral disorders and brain edema *in vivo* (Li et al., 2018). Hypoxia-pretreated olfactory mucosal mesenchymal stem cells (OM-MSCs) decreased the expressions of NLRP3, caspase-1, GSDMD, and IL-1β in microglia, reduced the microglial membrane pores, and inhibited microglial pyroptosis (Liu et al., 2021). Our team found that Didymin could suppress microglia pyroptosis and neuroinflammation by interrupting the assembly of NLRP3 inflammasome, and subsequently inhibiting Caspase-1/GSDMD mediated canonical pyroptotic signaling pathway after ICH (Gu et al., 2022).

### Inhibition of NLRP1/caspase-1/gasdermin D

Tang’s team used maraviroc (MVC), a selective antagonist of CCR5, to improve the short- and long-term neurobehavioral deficits and decreased neuronal pyroptosis in ipsilateral brain tissues at 24 h after ICH, which was accompanied by increased PKA-Cα and p-CREB expression, and decreased expression of NLRP1, ASC, C-caspase-1, GSDMD, and IL-1β/IL-18. Such effects of MVC were abolished by 666-15. They found that CCR5 activation promoted neuronal pyroptosis and neurological deficits after ICH in mice, partially through the CCR5/PKA/CREB/NLRP1 signaling pathway. CCR5 inhibition with MVC may provide a promising therapeutic approach to alleviate neuronal pyroptosis and neurological deficits in managing patients with ICH (Yan et al., 2021). Besides, they reported that RO27-3225 treatment, a selective agonist of MC4 receptor, could decrease neuronal pyroptosis and neurobehavioral deficits at 24 and 72 hr after ICH, with reduced expression of p-ASK1, p-JNK, p-p38 MAPK, NLRP1 inflammasome, cleaved caspase-1, and IL-1β after ICH. On the contrary, the specific MC4 receptor antagonist HS024 pretreatment prevented the effects of RO27-3225. Similar to RO27-3225, NQDI-1 alone improved neurological functions and down-regulated ASK1/JNK/p38MAPK expression after ICH. They provided a treatment that activation of MC4R by inhibiting the signaling pathway of ASK1/JNK/p38MAPK dependent on NLRP1 to improve neurologic impairment after ICH-induced pyroptosis (Chen et al., 2019c).

### Inhibition of NLRC4/caspase-1/gasdermin D

Gan et al’s team found that the expression of phosphorylated NLRC4 was increased after ICH, peaking approximately at 72 h after ICH, accompanied by the increased MPO, TNF-α, IL-6, caspase-1, IL-1β, and IL-18, while knockdown of NLRC4 reversed the expression of inflammatory cytokines, and in the meantime, neutrophil infiltration, neuronal cell death and blood-brain barrier disruption were alleviated. Besides, an LRRK2 inhibitor (GNE7915) was injected into the abdominal cavity. Short hairpin (sh) RNA lentiviruses and lentiviruses containing RGS2 were designed and applied to knock down and promote RGS2 expression. RGS2 suppressed the interaction of LRRK2 and NLRC4 and NLRC4 inflammasome activation by regulating pLRRK2. They demonstrated that RGS2/LRRK2 may relieve pyroptosis-mediated brain injury following ICH by restraining the NLRC4 inflammasome activation-dependent pyroptotic pathway (Gan et al., 2021).

### Others

The endoplasmic reticulum (ER) is the main organelle responsible for protein folding and assembly and the accumulation of wrongly folded proteins leads to ER stress while persisting or excessive ER stress can induce cell damage (Tabas and Ron, 2011). Previous studies have reported that ER stress can activate the NLRP3 inflammasome, which is thought to be a novel mechanism of atherosclerosis (Chen et al., 2019d). ER stress has also been found to induce pyroptosis in chronic liver disease and ischemia-reperfusion insult (Yang et al., 2014; Lebeaupin et al., 2015). Chen et al’s team investigated the role of ER stress in evoking neuronal pyroptosis and related mechanisms in a mouse ICH model. They used tauroursodeoxycholic acid (TUDCA) to inhibit ER stress and observed that TUDCA reduces neuronal pyroptosis and has a neuroprotective role. Besides, they found that ER stress inhibition alleviates neuronal pyroptosis by decreasing the expression of IL-13 after ICH, pointing to IL-13 as a novel therapeutic target in ER stress-induced neuronal pyroptosis after ICH (Wang Z. Z. et al., 2020).

Except for interrupting the information of inflammasome, there are some trails to inhibit the caspase protein, the key factor of pyroptosis. Liang’s team injected Ac-YVAD-cmk into the ICH model to inhibit the activation of pro-caspase-1 and found that the behavioral performance was improved, brain edema was alleviated, in association with decreasing activated microglia, and the expression of pyroptosis-related factors at 24 h post-ICH (Liang et al., 2019). Our team adopted VX-765 to inhibit caspase-1 and found that the neurological deficits and pyroptosis were attenuated following ICH.

Gasdermin D act as an indispensable executor in the process of pyroptosis, most of all, GSDMD, as a common final effector of pyroptotic death execution downstream of caspase-1 (canonical inflammasome) and caspase-11 (non-canonical inflammasome), is involved in the regulation of pyroptosis in multiple CNS disease (Xu et al., 2021b). Inhibiting GSDMD, therefore, represents a promising approach to treating inflammatory disorders. A recent study has reported that both endogenous fumarate and exogenously delivered dimethyl fumarate (DMF) convert the cysteines in GSDMD to S-(2-succinyl)-cysteines (a process called succinate) to prevent its interaction with caspases and subsequent processing and activation. Administration of DMF to mice alleviated inflammation in models of multiple sclerosis and familial Mediterranean fever, which indicated that GSDMD succinate prevents its interaction with caspases, limiting its processing, oligomerization, and capacity to induce cell death. These findings explained the efficacy of DMF as a treatment by directly interrupting the activation of GSDMD for multiple sclerosis and other inflammatory diseases and offer insights into future anti-inflammatory drug design (Humphries et al., 2020). Besides, Hu et al. identified disulfiram as a potent inhibitor of GSDMD pore formation. In cells, disulfiram inhibited cytokine release and prevented pyroptosis. In mice, the drug is protected from lethal lipopolysaccharide-induced septic shock (Crunkhorn, 2020; Hu et al., 2020). These discoveries above focused on GSDMD’s potential as a druggable target, then recent efforts in the development of inhibitors to interfere with the pore-forming function of GSDMD and thus alleviate the detrimental effects due to pyroptotic cell death. There aren’t any studies, though, testing GSDMs inhibitor as a post-ICH therapy in an animal model.

## Discussion

Over decades, increasing preclinical trials targeting SBI after ICH have been tried, and several potentially promising targets have been explored in animal ICH models with varying degrees of success. However, no prospective candidate target has been examined successfully in the clinical trial. Therefore, it is very necessary to further explore the molecular mechanism of SBI following ICH. Recent research has elucidated the role of microglial pyroptosis versus microglial neuroinflammation in ICH-induced brain injury and neurological deficits (McKenzie et al., 2020). Many preclinical studies in animal model experiments reported that inhibition of microglial pyroptosis could reduce perihematomal edema, attenuate neurologic deficits, and promote recovery of neurofunction. This review contributes to a better understanding of the function of microglial pyroptosis in ICH and its potential as a therapeutic target.

**FIGURE 4:**
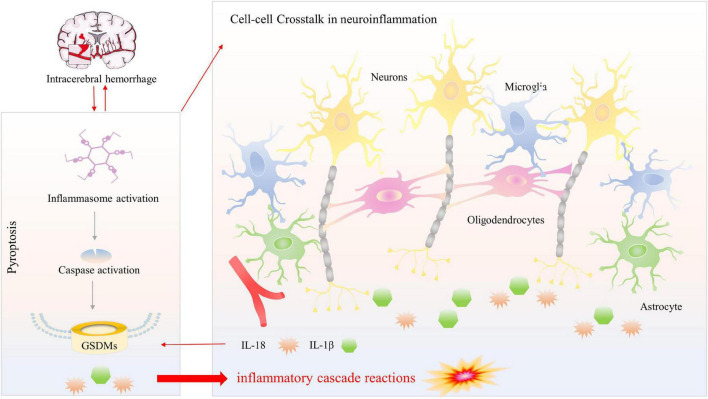
Pyroptosis versus neuroinflammation among multiple CNS cells. Activation of inflammasome following ICH promotes communication between CNS cells, that different signals crosstalk affect neuroinflammation and produce inflammatory cytokines (IL-1β and IL-18). Meanwhile, inflammasome activation results in caspase-1-mediated cleavage of GSDMD. N-GSDMD transformed into the cellular membrane to form non-selective pores, leading to the subsequent release of the mature inflammatory cytokines, causing magnify neuroinflammation and generating inflammatory cascade reactions.

This study has certain limitations, including the fact that we concentrated only on the mechanism and signaling route of microglial pyroptosis rather than on actual intercellular communication centered on CNS cells, a current research hotspot. In general, microglia, the most important immune sentinels, gather in the peri-hematoma, where they are the first inflammatory response cells. From there, they engage in cell-cell interactions with other elements of the neurovascular unit (NVU) (Tschoe et al., 2020). To be specific, microglia could toward the polarization of the pro-inflammatory M1-phenotype or anti-inflammatory M2-phenotype (Taylor and Sansing, 2013). Emerging evidence suggests that microglia are vital to neuronal homeostasis and may influence neuron destiny and that injured neurons control microglia activity *via* several signals (Suzumura, 2013; Peng et al., 2021). Astrocytes, like microglia, could polarize toward A1 phenotype or A2 phenotype under the influence of microglia after brain injury and disease (Linnerbauer et al., 2020), and meanwhile, it could influence the behavior of microglia (Vainchtein and Molofsky, 2020). Oligodendrocytes differentiation and enhancing myelination are essential after stroke, and microglia-derived cytokines can regulate oligodendrocyte differentiation and myelination (Zhang et al., 2013; Xie et al., 2021). Conversations among these CNS cells form feedback loops to participate in the secondary progression of neuroinflammation and pyroptosis after intracerebral hemorrhage (Figure 4). Therefore, to provide an experimental foundation and more effective therapeutic methods for the treatment of SBI, we must continue to investigate both the regulation mechanism of microglial pyroptosis and the interplay among CNS residents and invading cells after ICH.

## Conclusion

In conclusion, microglial pyroptosis is a major contributor to ICH-induced brain damage. Because there are no viable treatments for SBI, we decided to look into the translational potential of our results. Several significant papers have established that therapeutic drugs targeting key proteins in the pyroptotic pathway can improve neurological defects after ICH to a certain extent, but many studies focused on the pathway on the inflammasome assembly and caspase activation, and there are few studies on therapeutic drugs targeted at the gasdermin family, the ultimate executor of pyroptosis. Inhibiting pyroptosis by aiming at GSDMs protein could effectively block the release of pyroptotic molecules and excessive inflammatory factors.

## Author contributions

LG and ZX led the conceptualized study. ZX, YT, and XL revised it critically for important intellectual content. RL, XZ, and YY searched for documents about this topic. LG and MS wrote the first draft. ZX reviewed and edited the final draft. All authors contributed to the article and approved the submitted version.

## Abbreviations

BBB: blood-brain barrier
ECs: endothelial cells
CNS: central nervous system
ASC: apoptosis-associated speck-like protein containing a caspase recruitment domain
CARD: caspase recruitment domains
NLR: nucleotide-binding domain leucine-rich repeat-containing gene family
AIM2: absent in melanoma 2
PAMPs: pathogen-associated molecular patterns
DAMPs: damage-associated molecular patterns
TIRs: toll-like receptors
IL: interleukin
NF- κ B: nuclear factor kappa B
GSDMD: gasdermin D
GSDMD-NT: N-terminal fragment of GSDMD
IL-1 β: interleukin-1 β
IL-18: interleukin-18
ICH: intracerebral hemorrhage
SBI: secondary brain injury.
