# Supplementary material for: Microglial pyroptosis: Therapeutic target in secondary brain injury following intracerebral hemorrhage
Source: Front Cell Neurosci. 2022 Sep 9;16:971469. doi: 10.3389/fncel.2022.971469 (PMC9507402; doi:10.3389/fncel.2022.971469)
Supplement: Supplementary file 1 [file Data_Sheet_1.PDF]

Table 1: Pyroptosis-related inflammasome in ICH

Inflammasome	Expression after ICH	Location in brain	Mechanism	Reference
NLRP1	Increase at 3h, reach the peak at 24h, and lasting at 72h after ICH	Neuron, Astrocyte	CASP1/GSDMD canonical pathway of pyroptosis	(Chen et al., 2019c; Yan et al., 2021 ; (Schroder and Tschopp, 2010; Chavarria-Smith and Vance, 2015)
NLRP3	Increase at 3h, reach the peak at 24h after ICH	Neuron, Astrocyte, Microglia, Vascular endothelial cells	CASP1/GSDMD canonical pathway of pyroptosis	(Ma et al., 2014; Feng et al., 2015; Dong et al., 2016)
NLRC4	Increase after ICH, 3h, reach the peak at 72h after ICH	Astrocyte, Microglia	CASP1/GSDMD canonical pathway of pyroptosis<br><br>CASP8/ TLR/Nf-kB	(Freeman et al., 2017; Gan et al., 2021)
NLRP6	Increase at 6h, reach the peak at 24h, and lasting at 72h after ICH	Neuron, Astrocyte, Microglia	Unclear	(Wang et al., 2017a; Xiao et al., 2020)
